# Supplementary material for: Analysis of PPARγ Signaling Activity in Psoriasis
Source: Int J Mol Sci. 2021 Aug 10;22(16):8603. doi: 10.3390/ijms22168603 (PMC8395241; doi:10.3390/ijms22168603)
Supplement: Supplementary file 1 [file ijms-22-08603-s001.zip › Supplemental materials_Analysis of PPARg signaling activity in psoriasis/Pathway models/Models images and html files/PPARG expressed in cells assotiated with psoriasis/116153.html]

skin


# Organ skin

|  |  |
| --- | --- |
| URN | urn:agi-ncimorgan:C0684084 |
| Total Entities | 12 |
| Connectivity | 16232 |
| Name | skin |

---

|  |  |
| --- | --- |
| ChildConcepts | dermis |
|  | epidermis |
|  | epidermal ridge |
|  | node of Bizzozero |
|  | papillary area |
|  | scalp |
|  | skin cell |
|  | dander |
|  | glabrous skin |
|  | skin nerve |
|  | skin receptor |
|  | skin surface |

---

|  |  |
| --- | --- |
| Pathway | PPARG expressed in cells assotiated with psoriasis |
|  | Neighbors of skin |

---

|  |  |
| --- | --- |
| MedScan ID | 8800864 |

---

|  |  |
| --- | --- |
| Alias | skin layer |
|  | cutis |
|  | dermal |
|  | derma |
|  | skins |
|  | cutaneous |
|  | skin |
|  | skin layers |

---

|  |  |
| --- | --- |
| NCIm ID | C0684084 |

---
